# T-Cell-Replete Versus *ex vivo* T-Cell-Depleted Haploidentical Haematopoietic Stem Cell Transplantation in Children With Acute Lymphoblastic Leukaemia and Other Haematological Malignancies

**DOI:** 10.3389/fped.2021.794541

**Published:** 2021-12-24

**Authors:** Katharina Kleinschmidt, Meng Lv, Asaf Yanir, Julia Palma, Peter Lang, Matthias Eyrich

**Affiliations:** ^1^Department of Pediatric Hematology, Oncology and Stem Cell Transplantation, University Hospital of Regensburg, Regensburg, Germany; ^2^Beijing Key Laboratory of Hematopoietic Stem Cell Transplantation, National Clinical Research Center for Hematologic Disease, Peking University Institute of Hematology, Peking University People's Hospital, Beijing, China; ^3^Bone Marrow Transplant Unit, Division of Haematology and Oncology, Schneider Children's Medical Center of Israel, Petach-Tikva, Israel; ^4^The Sackler Faculty of Medicine, Tel-Aviv University, Tel-Aviv, Israel; ^5^Bone Marrow Transplant Unit, Hospital Dr. Luis Calvo Mackenna, Santiago, Chile; ^6^Department of Pediatric Hematology and Oncology, University Children's Hospital, University of Tuebingen, Tuebingen, Germany; ^7^Department of Paediatric Haematology, Oncology and Stem Cell Transplantation, University Children's Hospital, University Medical Center, University of Würzburg, Würzburg, Germany

**Keywords:** acute leukaemia, paediatric [MeSH], stem cell transplantation (HSCT), haploidentical allogeneic stem cell transplantation, T-cell depletion, post-transplant cyclophosphamide, Beijing

## Abstract

Allogeneic haematopoietic stem cell transplantation (HSCT) represents a potentially curative option for children with high-risk or refractory/relapsed leukaemias. Traditional donor hierarchy favours a human leukocyte antigen (HLA)-matched sibling donor (MSD) over an HLA-matched unrelated donor (MUD), followed by alternative donors such as haploidentical donors or unrelated cord blood. However, haploidentical HSCT (hHSCT) may be entailed with significant advantages: besides a potentially increased graft-vs.-leukaemia effect, the immediate availability of a relative as well as the possibility of a second donation for additional cellular therapies may impact on outcome. The key question in hHSCT is how, and how deeply, to deplete donor T-cells. More T cells in the graft confer faster immune reconstitution with consecutively lower infection rates, however, greater numbers of T-cells might be associated with higher rates of graft-vs.-host disease (GvHD). Two different methods for reduction of alloreactivity have been established: *in vivo* T-cell suppression and *ex vivo* T-cell depletion (TCD). *Ex vivo* TCD of the graft uses either positive selection or negative depletion of graft cells before infusion. In contrast, T-cell-repleted grafts consisting of non-manipulated bone marrow or peripheral blood graft*s* require intense *in vivo* GvHD prophylaxis. There are two major T-cell replete protocols: one is based on post-transplantation cyclophosphamide (PTCy), while the other is based on anti-thymocyte globulin (ATG; Beijing protocol). Published data do not show an unequivocal benefit for one of these three platforms in terms of overall survival, non-relapse mortality or disease recurrence. In this review, we discuss the pros and cons of these three different approaches to hHSCT with an emphasis on the significance of the existing data for children with acute lymphoblastic leukaemia.

## Introduction to Different Platforms for Haploidentical HSCT

Allogeneic haematopoietic stem cell transplantation (HSCT) represents a potentially curative option for children with high-risk or refractory/relapsed leukaemias. The use of fully haplotype mismatched haploidentical family donors has become an accepted option for patients who lack a matched related donor or matched unrelated donor (MUD) (1). Graft-vs.-leukaemia effects based on natural killer (NK)-cell alloreactivity have been observed in this setting in both children and adults (2, 3). Moreover, the easier availability of haploidentical donors offers the possibility to administer stem cell boosts, cellular therapies with anti-leukaemic effector cells or antigen-specific T cells or even to produce a second stem cell graft within a very short time (4, 5).

Transplantation of grafts from a donor who is fully haplotype mismatched causes profound bidirectional alloreactivity, both in the graft-vs.-host and the host-vs.-graft direction. To overcome these double barriers, different strategies have been developed:

- *ex vivo* graft manipulation procedures for T-cell depletion (TCD)
- *in vivo* TCD utilising post-transplant cyclophosphamide (PTCy)
- Use of unmanipulated grafts with intensive immune suppression regimens in combination with serotherapy (e.g., the Beijing protocol).

*Ex vivo* graft manipulation methods have evolved in recent years. CD34-positive selection of peripheral stem cells was the original standard practise in the early days of hHSCT; this technique minimised GvHD by effective reduction of T cells in the graft (6) but was accompanied by a high rate of infectious complications and endothelial damage. In order to reduce non-relapse mortality, TCD was refined by using CD3- and CD19-coated microbeads for depletion of T and B cells or with intravenous rituximab was established. With T- and B-cell depletion instead of CD34-positive selection, other immune components such as NK cells, dendritic cells and monocytes remained within the graft and could be used to generate anti-leukaemic, anti-viral or graft-facilitating effects (7, 8). Recently, a third procedure has gained wide acceptance due to its reliability and efficacy: T-cell receptor antibody (TCRAb) depletion, which selectively removes αβ^+^ T lymphocytes. This technique retains not only NK cells and other cells in the graft but also γδ^+^ T lymphocytes (9).

In addition to *ex vivo* procedures, *in vivo* T-cell depletion with application of PTCy has been established mainly in adult centres, with a high number of patients treated to date. Promising results have been reported in several trials (10). Since alloreactive T cells are depleted by cyclophosphamide *in vivo*, unmanipulated T-replete grafts can be given and no good manufacturing practise (GMP)-grade graft manipulation procedures are necessary. A third option is to use a combination of G-CSF primed bone marrow and PBSCs, thus an unmanipulated, T-cell repleted graft containing haematopoietic stem cells from two different sources. In order to suppress GvHD, intensive pharmacological immune suppression is applied: (i) anti-thymocyte globulin (ATG) immediately prior to transplant (thus extending its effect on donor T cells), (ii) MTX (45 mg/m2) followed by cyclosporine A plus MMF for an extended time period. This approach has been mainly described by Italian and Chinese groups; most data are available for the Beijing protocol (11–15).

Randomised trials comparing these three ways to deplete alloreactive T cells are still lacking. Having this drawback in mind, we searched existing medical data bases for studies including the key works “haploidentical haematopoietic stem cell transplantation,” “paediatric/childhood,” and “acute lymphoblastic leukaemia.” For analysis of clinical outcome parameters, we choose studies not older than 10-years of any phase (I–III). For proof-of-principle studies, also older publications and studies including animal models were acceptable. Despite the vast majority of available paediatric trials included children with diverse haematological malignanices, we tried to focus on outcome parameters of ALL cases, although a clear distinction is not always possible here. Therefore, some of our findings just represent an approximation to pure ALL data and do not exceed an evidence level of IIb or III. Nevertheless, our review represents the currently available data which can provide guidance to interested transplant physicians and help to design more focused and controlled clinical trials in this field.

## *Ex Vivo* T-Cell Depletion

Transplant numbers using hHSCT are constantly on the rise worldwide (16). Although new concepts such as *in vivo* PTCy have fostered the use and applicability of this approach, *ex vivo* TCD still represents the most reliable way to minimise alloreactivity: this technique enables a post-transplant course without significant immunosuppression yet with low rates of chronic GvHD (cGvHD).

Techniques for *ex vivo* TCD of the graft have evolved over time (17). The first successful endeavours in the clinical setting were undertaken in the early 1980s in children with primary immunodeficiencies (18). These ground-breaking early clinical trials using TCD with soybean agglutination of stem cells followed by rosette formation of human T cells with red blood cells from sheep helped to define requirements for engraftment and the maximum number of tolerated T cells in hHSCT (19). The discovery of the “megadose” concept (i.e., transplantation of more than 10^7^ haematopoietic stem cells /kg body weight of the recipient) in preclinical animal models (20) and parallel advances in stem cell selection techniques (21) paved the way for hHSCT with more standardised and large-scale TCD techniques. Indeed, the first clinical trials in leukaemia patients receiving large doses of highly purified, CD34-positively selected, haploidentical haematopoietic stem cells virtually devoid of any donor T cells confirmed the feasibility of the megadose concept in humans but pointed towards the Achilles' heel of hHSCT: the increased incidence of potentially lethal infectious complications (22). This susceptibility for overwhelming infections is caused by slow immune reconstitution after TCD hHSCT (23) (a result of the transfer of only minimal numbers of T-cell precursors with viral specificities) and, consequently, the reliance on age-dependent thymopoiesis (24, 25). These data were consistent with the clinical observation that adults with lower thymic capacity experienced more infectious complications than children.

The next generation of large-scale TCD techniques (CD3^+^/CD19^+^ depletion) replaced positive selection of haematopoietic progenitor cells by depletion of CD3^+^ T cells, thereby increasing the number of accessory cells such as monocytes, dendritic cells and NK cells in the graft. Although generally feasible, this approach failed to demonstrate clinical superiority in adult patients with advanced haematological malignancies: non-relapse mortality (NRM) was 42% 2-years after CD3^+^/CD19^+^ T-cell-depleted hHSCT, which was comparable to the 40% reported after CD34-selected transplantations (22, 26). Mortality due to infection in both landmark trials of CD34-selected vs. CD3^+^/CD19^+^ T-cell-depleted hHSCT was 26%. Furthermore, acute GvHD (aGvHD) and cGvHD in both adult and paediatric patient cohorts with advanced haematological malignancies after CD3^+^/CD19^+^ TCD was observed (8, 26), probably related to the 1-log lower TCD efficacy of this procedure compared to CD34-selection (27). Data on immune reconstitution were inconsistent. Whereas, one centre reported a faster reconstitution of CD3^+^ T cells after CD3^+^/CD19^+^ TCD positive stem cell selection in children with advanced haematological malignancies (28), another paediatric study in children with acute leukemias found differences in T-cell reconstitution to be more closely related to the type of conditioning rather than to the TCD technique used (29).

Meanwhile, more-refined mouse allotransplant models demonstrated that GvHD-inducing potential is primarily contained within the naïve donor T-cell pool carrying the αβ TCR (αβ T cells) (30), whereas T cells carrying the γδ TCR (γδ T cells) have little alloreactivity (31). Moreover, in a cohort of 153 patients with acute leukemias after partially matched HSCT (comprising both children and adults) those with higher than normal γδ-T-cell reconstitution showed significantly better 5-year leukaemia-free survival (LFS) and overall survival (OS) but no increased GvHD incidence (32), indicating that γδ T cells might contribute to immune control over leukaemia and infectious agents in humans. These observations led to development of a novel TCD approach—TCRαβ/CD19^+^ depletion—which selectively depletes αβ T cells from the graft yet retains large numbers of γδ T cells in addition to all other accessory cells. The excellent technical performance of that approach has been demonstrated (27, 33) and clinical feasibility was shown in several trials and case reports (34–44).

## Post-Transplant Cyclophosphamide

The introduction of PTCy as a platform for performing *in vivo* T-cell depleted hHSCT has revolutionised the field of clinical HSCT in the last decade. The availability and simplicity of this method, combined with its low cost, have made hHSCT-PTCy feasible to implement. It is a novel transplant platform for children with ALL and an HSCT indication (45).

Cyclophosphamide-induced allogeneic tolerance is thought to be mediated mainly by selective killing and suppression of proliferating alloreactive T cells. On days +3 and +4 after HSCT, alloreactive T cells are in their peak proliferative state, making them particularly sensitive to cyclophosphamide-mediated killing. Other resting memory and regulatory T cells (T_reg_) are relatively resistant to cyclophosphamide-induced killing, allowing them to survive and provide the recipient with immunity against infections (46) until a new T-cell repertoire can be produced. The stem cell component in the graft is also highly resistant to cyclophosphamide due to the activity of aldehyde dehydrogenase (47), which actively keeps cyclophosphamide out of the stem cells. This makes cyclophosphamide-induced killing highly specific for both donor-derived and recipient-derived alloreactive T cells, and an ideal way to induce tolerance in the allogeneic setting.

The John Hopkin's University group who pioneered this method based their clinical trials on pre-clinical data that demonstrated the ability of cyclophosphamide to eliminate allogeneic immune reactions, especially when given 2–3 days after allogeneic exposure (48–50). Early clinical trials were conducted in the early 2000s in patients with high-risk haematological malignancies using a non-myeloablative conditioning regimen. The initial protocol used bone marrow as the graft source. Cyclophosphamide 100 mg/kg was divided into 2 doses on days +3 and +4. The results were published in 2010 (51), and, since then, this platform has been adopted by many centres. Some centres have modified the original Hopkin's protocol to utilise different conditioning regimens (e.g., with myeloablative agents) (52) or different stem cell sources, including peripheral blood (53) or a combination of bone marrow and peripheral blood, for use in HSCT for many other malignant and non-malignant diseases, such as lymphoma (54) and severe aplastic anaemia (55). The efficacy of this method in preventing steroid-refractory GvHD has led to comparative studies that showed similar outcomes with hHSCT-PTCy vs. HSCT using matched donors (56), including matched sibling donors (MSDs) (57).

As HSCT-PTCy gained widespread use, specific reports regarding its use in ALL emerged. Srour et al. reported that 109 adult ALL patients who received HSCT-PTCy showed comparable outcomes to those who received historical standard human-leukocyte-antigen (HLA)-matched HSCT (58). The European Society for Blood and Marrow Transplantation (EBMT) has published a registry of HSCT-PTCy in 336 adult ALL patients, demonstrating 2-year LFS of 40%, which is better than a “traditional” hHSCT platform using ATG (*N* = 98) that resulted in 2-year LFS of only 24% (59). It is worth noting that most of the studies have focused on GvHD occurrence and disease control, and data regarding infections and other morbidities are lacking.

The available data on the efficacy and safety of hHSCT-PTCy for paediatric patients with ALL are scant and until recently were based mainly on a single centre's experience, reported together with other malignant diseases. This makes the task of drawing conclusions regarding use of PTCy in paediatric ALL challenging. More recently, Ruggeri et al. reported the EBMT registry data of 180 paediatric ALL patients transplanted from a haploidentical donor using PTCy (60). Although this study was retrospective and included a heterogeneous population and conditioning regimens, thus would still be considered a low evidence level, it still provides for the first time data on a relatively large population of paediatric ALL patients. Together with some small series, it enables us to review the current data on hHSCT-PTCy in paediatric patients with ALL and compare it with other platforms for hHSCT. These comparisons are the purpose of this review and are detailed subsequently.

## The Beijing Protocol

In 2019, ALL (*n* = 2,294) accounted for 24% of all allogeneic HSCT cases in China and was the second most prevalent indication. The rapid growth of allogeneic HSCT is a result of the increased availability of alternative donors, especially haploidentical donors. A total of 94% of HSCTs in China follow the Beijing Protocol in 2019, which comprises T-replete hHSCT with high-dose ATG and strengthened immune suppression (mycophenolate, ciclosporin, and methotrexate) with G-CSF mobilised bone marrow and/or peripheral blood while PTCy (Baltimore protocol uses high-dose post-transplantation cyclophosphamide on the third and fourth day after the transplant with other immune suppression (ciclosporin, mycophenolate mofetil, etc.) (61). Busulfan is not essential part of the Beijing protocol, there are also TBI-based conditioning regimens without Busulfan following Beijing protocol. We have defined Beijing protocol as high-dose ATG and strengthened immune suppression with G-CSF mobilised grafts. In China, haploidentical donors have been the largest source of allogeneic HSCT donors since 2013 and their prevalence among all donors increased to more than 60% in 2019. Other types of donors include MSDs (21.7%), unrelated donors (12.8%) and cord blood donors (5.4%) (62).

The Beijing protocol has proven superiority above chemotherapy in high-risk leukemias as consolidation therapy in first complete remission. For 104 paediatric patients with very high-risk Philadelphia chromosome (Ph)-negative B-cell ALL in first complete remission (CR1), hHSCT using the Beijing Protocol reduced the cumulative incidence of relapse (CIR) (10.9% vs. 46.7%, respectively; *p* < 0.001) and improved the LFS rate (81.0% vs. 52.0%, respectively; *p* = 0.005) compared with chemotherapy (15). For 68 paediatric Ph^+^ ALL patients, hHSCT using the Beijing Protocol improved the OS and LFS rates and the CIR in this high-risk group compared with imatinib plus intensive chemotherapy (63). In 37 children >1-year old with *KMT*2*A*^+^ B-cell ALL in CR1, hHSCT using the Beijing Protocol has been shown to improve LFS (89.5 % vs. 52.2 %, respectively, *p* < 0.001) and reduce CIR (5.3 % vs. 74.1 %, respectively; *p* < 0.001) compared with no HSCT (64). In 150 paediatric patients who had minimal-residual disease (MRD) recurrence (≥0.01%), Wang et al. demonstrated that hHSCT using the Beijing Protocol resulted in a lower 2-year CIR (23.3% vs. 64.0%, respectively; *p* < 0.001) and a higher OS rate (88.7% vs. 46.3%, respectively; *p* < 0.001) than did chemotherapy (65). Xu et al. reported that 48 children with high-risk T-cell ALL who received hHSCT using the Beijing Protocol during CR1 exhibited higher LFS (65.7% vs. 26.0%, respectively; *p* = 0.008) and a lower relapse rate (19.8% vs. 56.7%, respectively; *p* = 0.014) than did patients transplanted when not in CR1, indicating that paediatric patients with T-cell ALL in CR1 benefit from HSCT (66).

Furthermore, hHSCT with the Beijing protocol as conditioning regimen was compared to conventional HSCT from matched related or unrelated donors. In a Phase III biologically randomised multicentre study, Wang et al. compared patients with Ph^−^ high-risk ALL receiving hHSCT with the Beijing Protocol (*n* = 103) with those receiving MSD-HSCT (*n* = 83) (14). There were no differences in 3-year disease-free survival (DFS, 61% vs. 60%, respectively; *p* = 0.91) in CR, 3-year OS (68% vs. 64%, respectively; *p* = 0.56) from HSCT, treatment-related mortality (TRM, 13% vs. 11%, *p* = 0.84), or CIR (18% vs. 24%, *p* = 0.30) between donor types. Therefore, hHSCT is a valid alternative to post-remission treatment for high- and standard-risk adult patients with ALL in CR1 who lack an identical donor (14).

Han et al. retrospectively investigated the outcomes of hHSCT using the Beijing Protocol in adults with standard-risk Ph^−^ ALL in CR1 and compared these to outcomes for patients receiving an HSCT from an MSD or MUD. A total of 127 haploidentical, 144 MSD, and 77 MUD HSCT recipients were included in the study. There were no differences in the rate of grade III–IV aGvHD (11.4% vs. 7.7% vs. 13.5%, respectively; *p* = 0.468), 5-year TRM (16.4% vs. 11.6% vs. 19.6%, respectively; *p* = 0.162), 5-year CIR (14.8% vs. 21.1% vs. 16.7%, respectively; *p* = 0.231), 5-year OS (70.1% vs. 73.7% vs. 69.8%, respectively; *p* = 0.525), 5-year DFS (68.7% vs. 67.3% vs. 63.7%, respectively; *p* = 0.606) or 3-year GvHD-free relapse-free survival (GRFS, 50.8% vs. 54.9% vs. 52.2%, respectively; *p* = 0.847) (67). In a recent prospective multicentre study of 131 young adults with standard-risk ALL who were in CR1 and did not have an HLA-matched donor, hHSCT using the Beijing Protocol was reported to result in a lower 2-year CIR (12.8% vs. 46.7%, respectively; *p* = 0.0017) and better 2-year DFS (80.9% vs. 51.1%, respectively; *p* = 0.0116) and OS (91.2% vs. 75.7%, respectively; *p* = 0.0408) than adult-dose chemotherapy (68). Consequently, hHSCT and MSD-HSCT are recommended equally as standard care in patients with high-risk and standard-risk Ph^+^-ALL in CR1.

Will an MSD always be the first donor choice for ALL? Possibly not. In a retrospective study of 82 Ph^+^ ALL (paediatric and adult) patients, hHSCT using the Beijing Protocol was associated with a significantly lower relapse rate than MSD-HSCT (44.8% vs. 19.1%, respectively; *p* < 0.05), with no differences in NRM, LFS, or OS between the two groups (69). In a Phase III biologically randomised trial of 208 patients (paediatric and adult) with MRD-positive ALL, hHSCT using the Beijing Protocol was associated with lower 3-year CIR (23% vs. 47%, respectively; *p* = 0.006) and higher LFS (65% vs. 43%, respectively; *p* = 0.023) and OS (68% vs. 46%, respectively; *p* = 0.039) than was MSD-HSCT. Multivariate analysis confirmed that hHSCT using the Beijing Protocol was the only factor affecting CIR, LFS and OS (70). In another retrospective study of 208 patients (paediatric and adult) with Ph^+^ ALL with positive pre-transplant MRD, hHSCT using the Beijing Protocol led to a lower 4-year CIR (14.8% vs. 56.4%, respectively; *p* = 0.021) and higher 4-year LFS (77.7% vs. 35.9%, respectively; *p* = 0.036) and OS (80.5% vs. 35.9%, respectively; *p* = 0.027) than did MSD-HSCT (71). These results suggest that hHSCT might be superior to MSD-HSCT in ALL patients with a high relapse risk.

A registry-based study compared the Beijing Protocol to PTCy in myeloablative hHSCT for haematologic malignancies. It included 220 patients, of whom 176 received hHSCT with the Beijing Protocol and 44 received hHSCT-PTCy; data were analysed using the nested case-pair method (1:4) to balance the disparity of age, diagnosis, status at HSCT, and tranplant year, etc. The incidences of 30-day neutrophil engraftment (88.6% vs. 96.6% in the PTCy group vs. Beijing protocol, respectively; *p* = 0.001) and 90-day platelet engraftment (84.1% vs. 94.2% in the PTCy group vs. Beijing protocol, respectively; *p* = 0.04) and the median time to neutrophil engraftment (17 days vs. 12 days in the PTCy group vs. Beijing protocol, respectively; *p* = 0.000) and platelet engraftment (22 days vs. 17 days in the PTCy group vs. Beijing protocol, respectively; *p* = 0.001) were significantly inferior in the PTCy group. The incidences of 30-day neutrophil engraftment (PT-CT vs. Beijing Protocol:88.6% vs. 96.6%, respectively; *p* = 0.001) and 90-day platelet engraftment (84.1% vs. 94.2%, respectively; *p* = 0.04) and the median time to neutrophil engraftment (17 days vs. 12 days, respectively; *p* = 0.000) and platelet engraftment (22 days vs. 17 days, respectively; *p* = 0.001) were significantly inferior in the PTCy group. The incidences of grade II–IV and III–IV aGVHD, cGVHD and severe cGVHD were comparable between arms. Patients in Beijing Protocol group had superior 3-year DFS (PT-CT vs. Beijing Protocol: 61.0% vs. 74.3%, respectively; *p* = 0.045) and OS (65.2% vs. 78.3%, respectively; *p* = 0.039) vs. those in the PTCy group (72).

In an EBMT registry analysis with a total of 308 patients, 193 received PTCy and 115 received ATG as GvHD prophylaxis. The incidence of grade II–IV aGVHD (31% vs. 21%, respectively; *p* = 0.07), 2-year chronic GvHD (33.7% vs. 28.3%, respectively; *p* = 0.33), relapse (21.6 vs. 22.3%, respectively; *p* = 0.97), NRM (22.4% vs. 30.5%, *p* = 0.19), LFS (56% vs. 47.2%, respectively; *p* = 0.26), and OS (58% vs. 54.2%, respectively; *p* = 0.37) were comparable between the PTCy and ATG groups (73).

## Comparing Outcomes with Different Haploidentical HSCT Platforms

Most retrospective series comparing outcomes with different protocols for hHSCT include all leukaemia types and ages together, making the task of drawing conclusions for paediatric ALL very challenging. Furthermore, most reports have used different types of conditioning—some chemotherapy-based and some total body irradiation (TBI)-based—which by itself has a very strong impact on outcomes. We will focus here on the publications which have specific data for paediatric patients with ALL, keeping in mind that true scientific comparison between the different haploidentical platforms in paediatric patients with ALL cannot be made based on existing data.

Reports of centres using the Beijing protocol have focused mainly on key transplant outcome parameters such as leukaemia-free and overall survival and incidences of acute and chronic GvHD, whereas data on other important transplant-related factors such immune reconstitution, viral reactivations and severe complication affecting NRM are still scarce. For full evaluation of this method, assessment of such data is essential.

## Relapse and Survival

Relapse and survival outcomes of key studies using different approaches to hHSCT are discussed below and summarised in Table 1 (15, 35, 60, 63, 66, 74). To simplify data presentation, we picked for each method only original reports with the best available data based on the ALL-population size and uniformity of the method.

**Table 1:** Summary of the largest studies assessing outcomes of haploidentical HSCT in paediatric patients with ALL, according to T-cell-depletion methodology used.

**Methodology**	**PTCy**	**Beijing Protocol**	***Ex vivo*** **T-cell depletion**	
Reference(s)	Ruggeri et al. (60)	Xue et al. (15), Xue et al. (63), Xu et al. (66)	Bertaina et al. (35)	Diaz et al. (74)
Type of study	Retrospective, registry based (EBMT, multicenter)	Retrospective, single center	Retrospective, multicenter	Prospective, observational, single center
ALL patients, *n*	180	B-cell ALL: 42 Ph^+^ ALL: 37 T-cell ALL: 48	68	28
Cumulative incidence of relapse	2-year CIR: CR1: 25% CR2: 37% CR3: 50%	3-year CIR: B-cell ALL: 11.9% Ph^+^ ALL: 15.9% T-cell ALL: 30.8%	5-year CIR: 29%	2-year CIR: 28%
Overall survival	2-year OS: CR1: 76% CR2: 61% CR3: NR	3-year: B-cell ALL: 81% Ph^+^ ALL: 87% T-cell ALL: NR	5-year OS: 68%	NR
Leukaemia-free survival	2-year LFS: CR1: 65% CR2: 44% CR3: NR	3-year LFS: B-cell ALL 81% Ph^+^ALL 77% T-cell ALL 54.4%	5-year LFS: 62%	2-year LFS: 36%

*ALL, acute lymphoblastic leukaemia; CIR, cumulative incidence of relapse; CR, complete remission; HSCT, haematopoietic stem cell transplantation; LFS, leukaemia-free survival; OS, overall survival; NR, not reported; Ph, Philadelphia chromosome*.

In a study of HSCT-PTCy in 180 paediatric patients with ALL (age range 0–18 years), Ruggeri et al. reported a 2-year CIR of 25, 37, and 50% for patients transplanted in CR1, CR2, and CR3, respectively (60). Two-year OS, LFS and GFRS were 50.8, 38.5, and 29.2%, respectively, for the whole cohort. When subdivided by CR status at time of transplant, OS and LFS were 65 and 76%, respectively, for patients in CR1, and 44 and 61%, respectively, for patients in CR2.

Other publications report varied incidences of relapse after transplantation (between 23 and 45%), but all of these studies included multiple types of haematological malignancy and had very few ALL patients transplanted using PTCy (75–78).

In studies using the Beijing protocol for hHSCT in paediatric patients (age range 2–17 years), the Beijing group reported a 3-year CIR of 11.9% in 42 patients with B-cell ALL (15), 15.9% in 37 patients with Ph^+^ ALL (15, 63), and 30.8% in 48 patients with T-cell ALL (66). The 3-year OS and LFS were 80.6 and 81%, respectively, for patients with B-cell ALL (15) and 87 and 77.2%, respectively, for those with Ph^+^ ALL (15, 63). Three-year LFS was 54.4% for patients with T-cell ALL (66) but data regarding OS was not provided by the authors.

In a study by Bertaina et al. of HSCT using *ex vivo* αβ TCD in 98 patients with acute leukaemia, 68 paediatric ALL patients were included. The estimated 5-year CIR for the whole group was 29%, and 5-year OS and LFS were 68 and 62%, respectively (35).

Diaz et al. reported a 2-years cumulative incidence of relapse of 28% in a group of 60 acute leukaemia patients treated with hHSCT using *ex vivo* αβ TCD of whom 28 had ALL (74). Two-year DFS for ALL patients only was 36%; this was significantly different from the 2-year DFS for patients with acute myeloid leukaemia (65%, *p* = 0.035).

Taken together, current data do not demonstrate the superiority of one method of hHSCT over the others, as no head-to-head superiority trials have been performed. Although, outcomes for B-cell ALL patients appear best when the Beijing protocol is used, it should be interpreted cautiously since some post HSCT parameters like immune reconstitution are not available. Furthermore, heterogenous patient populations, different trial methodologies and different supportive care approaches mean that no definitive conclusions can be drawn when comparing outcomes between trials. Only prospective randomised trials using defined patient populations, the same conditioning regimen and the same supportive care might reveal the superiority of one method over the others.

## Engraftment and Immune Reconstitution

With respect to engraftment, *ex vivo* TCD transplants have a significantly shorter interval to neutrophil engraftment than do PTCy transplants (>500/μL: 10 vs. 15 days, respectively) and a trend towards faster thrombocyte engraftment (>20,000/μL: 16 vs. 20 days, respectively) but lower rates of primary engraftment (88 vs. 100%, respectively) (79). However, in recent studies using TCRAb/CD19-depletion, hHSCT resulted in 96–98% primary engraftment rates after sufficiently intense immunosuppressive conditioning (35, 41, 80), indicating that both techniques yield comparable and safe engraftment with a faster neutrophil recovery after TCRAb/CD19-depletion.

In contrast, immune reconstitution after T-depleted vs. T-replete hHSCT shows striking differences. *Ex vivo* TCD results in a very early wave of immature NK cells in the absence of CD3^+^TCRαβ^+^ T cells during the first 2–3 months. This phase is followed by slow T- and B-cell reconstitution which starts in children after 3–4 months. Refinements in graft manipulation such as the selective removal of TCRαβ^+^ T cells have added an early wave of CD3^+^TCRγδ ^+^ T cells in parallel to NK-cell reconstitution. CD3^+^TCRγδ ^+^ T cells have only limited GvHD-inducing potential (31), thus their early appearance does not necessitate immunosuppression. However, they recognise cytomegalovirus (CMV) epitopes (81) and Epstein-Barr virus epitopes (82), as well as leukaemic cells (83) and solid tumours (84). This early availability of broadly reactive CD3^+^TCRγδ^+^ T cells most likely contributed to the significantly improved TRM rates of only 5–10% observed after TCRαβ-depleted hHSCT in children with acute leukaemias (35, 41, 43).

Patterns of immune reconstitution after PTCy resemble those of HLA-matched T-replete HSCTs. CD3^+^ T-cell numbers after hHSCT-PTCy have been reported to be similar to HSCTs from matched donors on day +30 (85) and day +100 (57), and higher than after T-cell depleted hHSCT, at least in the first 6 months (86, 87). Mechanistic studies in animal models have shown that PTCy selectively depletes T cells strongly stimulated by contact with allogeneic or exogenous antigens on days 0 to 2, whereas donor-derived naïve T cells undergoing slow homeostatic cycling are spared (88). This enables a relatively broad TCR repertoire of graft-derived T cells with the ability to react against pathogens at later stages. Shifting expression patterns from naïve to stem-like phenotypes results in a preponderance of stem cell memory T cells in the early phase of immune reconstitution after PTCy (89). However, the efficacy of PTCy in eliminating alloreactive clones and thereby preventing aGvHD and cGvHD seems to be less than with *in vitro* approaches of selective allodepletion, such as photodepletion techniques (90). One possible explanation for this is that TCRs with a lower affinity for alloantigens are not engaged in the hyperacute alloreaction on day +3 but, nevertheless, can give rise to GvHD responses at later stages. Occurrence of GvHD in PTCy transplants is influenced by circulating T_reg_ numbers: higher CD45RA^+^ T_reg_ numbers in the graft are associated with lower rates of aGvHD (91), while a higher ratio of T_reg_ to conventional T cells (T_con_) leads to lower incidences of cGvHD (92).

In conclusion, immune reconstitution after PTCy resembles patterns after T-replete transplants from matched donors. Application of cyclophosphamide on day +3 and +5 after transplantation depletes T-cell clonotypes with high affinity for recipient HLA or minor histocompatibility antigens; however, alloreactivity is not entirely eliminated. This is reflected by higher rates of aGvHD and cGvHD and also TRM compared with hHSCT using *ex vivo* TCD. Immune reconstitution after PTCy can partially be influenced by modulation of post-transplant immunosuppression and by choosing younger donors with a large naïve T-cell pool (91). No data on immune reconstitution after hHSCT using the Beijing protocol are available so far.

In TCD hHSCT, novel methods of improving immune reconstitution are currently under clinical evaluation, e.g., by filling the early T-cell compartment with pathogen-specific clonotypes. Adoptive transfer of low numbers of donor memory T cells has resulted in the occurrence of virus-specific responses in 65% of infused patients and a low rate of infectious complications without *de novo* GvHD (93). Thus, TCD hHSCT with state-of-the-art graft engineering results in more predictable immune reconstitution with preventable infections and better GvHD control.

## Graft-vs.-Host Disease

aGvHD continues to represent a major cause of transplant-associated morbidity and mortality after allogeneic HSCT. Whereas, in the adult transplantation setting GvHD rates up to 50% are tolerated by clinicians and considered “relatively low,” in the setting of paediatric HSCT the rate of high-grade aGvHD is aimed to be below 20%. Modern transplant regimens should prevent cGvHD at best completely, as the negative impact of long-term steroid therapy in the paediatric patient population is exhaustively known.

With hHSCT being increasingly used as an alternative therapeutic option, GvHD was one of the major initial concerns (94). *Ex vivo* TCD with continuously evolving selection techniques (CD34^+^ selection, CD3^+^/CD19^+^ depletion, and αβ/CD19^+^ depletion) proved to be a useful method to overcome the initially high GvHD rates (1, 17). However, for technical and economic reasons, *ex vivo* TCD is not ubiquitously available and the PTCy approach is widely used as an easily accessible platform for hHSCT.

The incidence of GvHD, however, is determined not only by choice of the transplant platform (e.g., PTCy vs. TCD vs. the Beijing protocol), but also by the stem cell source (bone marrow vs. peripheral blood stem cells [PBSC]) and the associated pharmacological GvHD prophylaxis (45, 95). In the literature, there is enormous heterogenicity among GvHD prophylactic regimens, even in the same treating centre. In particular, the application of different ATG forms (antithymocyte globulin; Thymoglobuline^®^ vs. Anti-T-lymphocyte globulin; ATG-Neovii^®^ represents a significant bias in data comparison due to the substantial differences in half-life duration (t_1/2_) and pharmacological mechanism of action (96–98). So is the t_1/2_ in case of ATG-Neovii^®^ significantly longer with a median time of 14 (4–45) days, when compared to Thymoglobuline^®^ with only 2–3 days of elimination t_1/2_. Moreover, even within the same platform, content of residual T-cells in the depleted grafts may vary which could impact on GvHD rates, jeopardising direct comparative analyses (35, 74). In T-cell depleted hHSCTs, a T-cell dose of 2.5 × 10^4^ CD3^+^/kg BW is considered safe as with this dose almost no GvHD could be observed (22). Recently, Bertaina et al. recommended not to exceed an upper limit of 1 × 10^5^ residual TCRαβ+ T cells/kg recipient body weight (35).

Reviewing the literature, however, some trends can be observed (see Table 2 for a summary) (9, 12, 14, 35, 41, 60, 74, 75, 78, 79, 95, 99–103). Generally, the cumulative incidence rates of severe (grade III–IV) aGvHD and overall as well as extensive cGvHD are higher in patients treated with the PTCy platform compared with those treated with *ex vivo* TCD (Table 1) (35, 95). Bertaina et al. showed in the largest cohort to date of paediatric patients (aged 0–21 years) with acute leukaemia who received hHSCT (*n* = 98) that low-grade skin aGvHD (grade I–II) is observed in up to 16% of patients receiving *ex vivo* TCD hHSCT; however, severe skin or gut GvHD did not occur at all (0%). Extensive cGvHD was a rare event with a cumulative incidence of 1% (35). Locatelli et al. reported similar data from a cohort of 80 paediatric patients affected by acute leukaemia who received an hHSCT after αβ T-cell and B-cell depletion: 30% of patients developed grade I–II aGvHD, but none developed higher grade aGvHD. cGvHD was reported in 5% of patients in absence of extensive forms (0%) (41).

**Table 2:** Cumulative incidence of GvHD following hHSCT in studies using PTCy, *ex vivo* TCD or the Beijing Protocol.

**References**	***N***	**Median age (range), years**	**HSCT indication**	**Disease status at HSCT**	**Stem cell source**	**Conditioning regimen**	**T cell depletion (*in vivo/ex vivo*)**	**Immuno-suppression (duration)**	**Median follow-up (range)**	**aGvHD**		**cGvHD**	
										**Grade II–IV**	**Grade III–IV**	**All**	**Extensive**
**Post-transplant cyclophosphamide**													
							**Cy dose mg/kg (days)**						
Ruggeri et al. (60)	180	9	ALL (100%)	CR1 (24%) CR2 (45%) >CR3 (12%) Active disease (19%)	BM (64%) PBSC (36%)	TBI-based (25.6%) MAC/chemother. (51.7%) RIC (22.7%)	50 (+3, +4)	CNI, MMF, MTX (no data on duration)	2.7-years	28.3%	12.4%	21.9%	9.5%
Katsanis et al. (99)	13	19.4 (4.6–26.1)	ALL (*n =* 7; 53.8%) AML (*n =* 3; 23.1%) Lymphoma (*n =* 2; 15.4%) Undifferentiated leukaemia (*n =* 1; 7.7%)	ALL: CR1 (*n =* 1; 14.3%) CR2 (*n =* 5; 71.4%) CR4 (*n =* 1; 14.3%)	No data	TBI-based (*n =* 7; 100% of ALL) Busulfan-based (*n =* 6; 100% of others)	69.2%: 50 (+3, +4); 15.4%: 50 (+3) and 40 (+4); 15.4%: 50 (+3) and 20 (+4)	MMF (28 d) + tacrolimus (median 149 d; range 95–222)	15.6 (1.5–31.2) months	30.8%	0%	23.1%	15.4%
Medina et al. (78)	52	9 (1.1–17)	ALL (61%) AML (25%) MDS (8%) CML (2%) NHL (2%) HL (2%)	Leukemia (*n =* 45): CR1 (42.2%) >CR2 (48.9%) Active disease (9.8%)	BM (60%) PBSC (40%)	Busulfan-based (*n =* 50)	50 (+3, +4)	81% CsA + MTX (no data) 19% CSA + MMF (no data)	No data	42%	8.5%	19%	No data
Trujillo et al. (100)	42	11 (2–17)	ALL (62%) AML (31%) JMML (5%) CML (2%)	CR1 (33%) CR2 (50%) CR3 (14%) Refractory (3%)	PBSC (100%)	TBI-based (100%)	50 (+3, +4)	MMF (60 d) + CsA (6 months)	45 months (surviving patients)	43%	17%	29%	No data
Berger et al. (75)	33	12 (1–21)	ALL (45%) AML (21%) Dendritic cell leukaemia (3%) MDS (12%) CML (3%) Lymphoma (HL and NHL) (15%)	CR1 (24.2%) CR2 (30.3%) CR3 (15.2%) Other (30.3%)	BM (91%) PBSC (9%)	MAC (42%) NMA (57%)	50 (+3, +4)	MMF (35 d) + 60.6% tacrolimus (180 d), 39.4% CsA (180 d)	383 (61–1,203) days	22%	3%	4%	No data
Hong et al. (101)	34	11.1 (0.9–20.3)	ALL (32.4%) AML (20.6%) MPAL (8.8%) Other malignant (5.9%) Non-malignant (32.4%)	CR1 (47.1%) >CR2 (20.6%) N/A (32.3%)	PBSC (100%)	Busulfan-based (100%)	50 (+3, +4)	MMF (35 d) + tacrolimus (8–12 months)	26 (1–50) months	38.2%	5.9%	No data	9.1%
Uygun et al. (102)	62	8.3 (0.4–20)	Malignant (63%) ALL (*n =* 22; 56.4%) AML (*n =* 7; 18.0%) sAML (*n =* 2; 5.1%) NHL (*n =* 3; 7.7%) MDS (*n =* 3; 7.7%) JMML (*n =* 2; 5.1%) Non-malignant (37%)	CR1 (28%) >CR2 (72%)	BM (31%) BM + PBSC (66%) PBSC (3%)	Busulfan-based (73%) TBI-based (3%) Other 24%)	47% 50 (+3, +4, +5); 53% (+4, +5)	35% CNI + Mp; 65% CNI (6–12 months) + MMF (1–3 months)	26 (6–57) months (survivors)	47%	No data	11%	5%
Dufort et al. (79)	23	15 (1–26)	ALL (*n =* 12; 52.2%) AML (*n =* 7; 30.4%) MDS (*n =* 3; 13.1%) LCH (*n =* 1; 4.3%)	CR1 (*n =* 8; 34.8%) CR2 (*n =* 7; 30.5%) CR3 (*n =* 2; 8.7%) Refractory (*n =* 3; 13%) Other (*n =* 3; 13%)	PBSC (100%)	MAC (16) RIC (7)	50 (+3, +4)	MMF (45 d) + CsA (90 d)	17 (7–76) months (survivors)	45%	5%	53%	12%
Perez-Martinez et al. (95)	41	6.64 (IQR 9.035)	ALL (58.5%) AML (24.4%) MDS (7.3%) JMML (2.4%) CML (2.4%) Biphenotypic (4.9%)	MRD in leukaemia: <0.01 (63.9%) >0.01 (36.1%)	PBSC (78%) BM (22%)	Busulfan-/melphalan-based (100%)	50 (+3, +4)	MMF + tacrolimus (4 months)	722 (IQR 914.5) days	52.6%	28.2%	47.7%	No data
***Ex vivo*** **T-cell depletion**													
							**Type of depletion**						
Bertaina et al. (35)	98	6.6 (0.1–17.3)	ALL (68%) AML (32%)	ALL: CR1 (28%) CR2 (57%) Other (15%) AML: CR1 (77%) CR2 (23%) Other (0%)	PBSC (100%)	TBI-based (74%) Busulfan-based (18%) Treosulfan-based (7%) Other (1%)	αβ/CD19 neg. selection	ATLG (no data)	3.3 (1.5–7.0, for surviving patients) years	16%	0%	6%	1%
Locatelli et al. (41)	80	9.7 (0.9–20.9)	ALL (70%) AML (30%)	ALL: CR1 (19%) CR2 (46%) >CR3 (5%) AML: CR1 (20%) CR2 (10%)	PBSC (100%)	TBI-based (75%) Busulfan-based (25%)	αβ/CD19 neg. selection	ATLG (d −5 to −3)	46 (26–60) months	30%	0%	5%	0%
Dufort et al. (79)	17	6 (0.5–17)	ALL (*n =* 5; 29.4%) AML (*n =* 6; 35.3%) JMML (*n =* 4; 23.5%) CML (*n =* 1; 5.9%) MDS (*n =* 1; 5.9%)	CR1 (*n =* 8; 47%) CR2 (*n =* 7; 41.2%) Other (*n =* 2; 11.8%)	PBSC (100%)	RIC (100%)	100%: CD3 neg. selection, 64.7%: additional CD34 pos. selection	CsA (30 d)	86 (39–128) months (survivors)	20%	7%	9%	9%
Perez-Martinez et al. (95)	151	9.05 (IQR: 7.92)	ALL (54.3%) AML (33.8%) MDS (6%) JMML (4.6%) Biphenotypic (1.3%)	MRD in leukaemia: <0.01 (62.4%) >0.01 (37.6%)	PBSC (100%)	Busulfan-/melphalan-based (most) Heterogenous (other centres)	54.3%: CD3/CD19 neg. selection; 22.5%: αβ/CD19 neg. selection; 14.6%: CD34+ purified and CD45RA naïve depleted; 8.6% CD34+ purified	CsA or MMF (30 d)	596 (IQR 1,203) days	30.6%	14.7%	28.6%	No data
Lang et al. (9)	41	9 (2–18)	ALL (*n =* 20; 48.8%) AML (*n =* 9; 21.9%) MDS/JMML (*n =* 3; 7.3%) Relapsed solid tumours (*n =* 4; 9.8%) Non-malignant (*n =* 5; 12.2%)	Malignancies (*n =* 36): First HSCT: CR1/CR2 (*n =* 6;16.7%) ≥CR3 (*n =* 4; 11.1%) Active disease (*n =* 4; 11.1%) Subsequent HSCT: CR1/CR2 (*n =* 6; 16.7%) ≥CR3 (*n =* 8; 22.2%) Active disease (*n =* 8; 22.2%)	PBSC (100%)	Melphalan-based (100%)	αβ/CD19 neg. selection	17%: OKT3 (d −8 to −1) 83% ATG-Fresenius (d −12 to −9)	1.6-years (survivors)	10%	15%	18%	9%
Diaz et al. (74)	60 (63 HSCT)	9 (1–19)	ALL (44%) AML (43%) MDS (8%) HL (3%) NHL (2%)	CR1 (36%) CR2 (32%) >CR3 (32%)	PBSC (100%)	Busulfan-based (100%)	αβ/CD19 neg. selection	CsA (until engraftment)	28 (4–72) months	34%	30%	25%	10%
**Beijing Protocol**													
Wang et al. (103)	756	25 (3–57)	AML (42.5%) ALL (39.5%) CML (18%)	AML: CR1 (*n =* 234; 30.9%) CR2 (*n =* 29; 3.8%) >CR3 (*n =* 5; 0.7%) Non-remission (*n =* 53; 7.0%)									
				ALL: CR1 (*n =* 183; 24.2%) CR2 (*n =* 38; 5.0%) >CR3 (*n =* 4; 0.5%) Non-remission (*n =* 14; 1.9%) Ph+ (*n =* 60; 8.0%) CML: First chronic phase (*n =* 77; 10.2%) Later chronic phase (*n =* 59; 7.8%)	BM + PBSC (100%)	Busulfan-based (100%)	n.a.	CsA (d −9 to 9 months) + MMF (d −9 to +60) + MTX (d +1, 3, 7; intervals of 7d max. 8 doses)	1,154 (335–3,511) days	43%	14%	53%	23%
Wang et al. (14)	103	26 (18–56)	ALL, high-risk (100%)	CR1 (100%)	BM + PBSC (100%)	Busulfan-based (100%)	n.a.	CsA + MMF + MTX (no data)	1,031 (370–1,638) days	28%	6%	38%	14%
Di Bartolomeo et al. (12)	80	37 (5–71)	ALL (*n =* 15; 18.8%) AML (*n =* 45; 56.2%) CML (*n =* 5; 6.2%) MDS (*n =* 3; 3.8%) HL (*n =* 5; 6.2%) NHL (*n =* 2; 2.5%) Plasma cell leukaemia (*n =* 3; 3.8%) Other (*n =* 2; 2.5%)	ALL (*n =* 15): CR1 (*n =* 8; 53.4%) CR2 (*n =* 2; 13.3%) >CR3 (*n =* 5; 33.3%) AML (*n =* 45): CR1 (*n =* 21; 46.7%) CR2 (*n =* 13; 28.9%) >CR3 (*n =* 11; 24.4%)	BM + PBSC (100%)	MAC (80%) RIC (20%)	n.a.	ATG Fresenius (d −4 to −1); CsA (d −7 to d 365); MTX (d +1, 3, 6, 11); MMF (d 7 to d 100); basiliximab (d 0, d 4)	18 (6–74) months	24%	5%	12%	5%

*aGvHD, acute graft-vs.-host disease; ALL, acute lymphoblastic leukaemia; AML, acute myeloid leukaemia; ATG, anti-thymocyte-globulin; BM, bone marrow; CML, chronic myeloid leukaemia; cGvHD, chronic graft-vs.-host disease; CR, complete remission; CsA, Cyclosporine A; Cy, Cyclophosphamide; HL, Hodgkin lymphoma; HSCT, haematopoietic stem cell transplantation; IQR, interquartile range; JMML, juvenile myelomonocytic leukaemia; LCH, Langerhans cell histocytosis; MAC, myeloablative conditioning; MDS, myelodysplastic syndrome; MMF, mycophenolate mofetil; MPAL, mixed-phenotype acute leukaemia; MTX, methotrexate; Ph, Philadelphia chromosome; NHL, non-Hodgkin lymphoma; PBMC, peripheral blood stem cells; RIC, reduced-intensity conditioning; sAML, secondary AML; TBI; total body irradiation*.

When comparing a/cGvHD rates with patients treated with Beijing protocol, moderate differences in the rates of acute GvHD can be observed with higher incidences in study populations including adult patients, and comparable GvHD rates for exclusively paediatric cohorts: Rates of grade III-IV aGvHD for Beijing protocol were reported to be 23.1% in a mixed adult/paediatric study population (13) and 17.1% in an exclusively paediatric patient cohort (63) compared with 12.4% in the largest paediatric study using PTCy (60) and 0% in the main paediatric study using *ex vivo* TCD (35); thus suggesting a role of the recipient's age impacting the development of GvHD. The differences with regard to cGvHD are more distinct: rates of extensive cGvHD of 23% (13, 103), 24.2% (63), and 28.4% (66) were observed in three studies using the Beijing protocol, which stand in contrast to rates of 9.5% with PTCy (60) and 1% using *ex vivo* TCD. Although the direct comparison of the studies is challenging due to different study designs (see Table 2), including differences in study population size, HSCT indication, disease status and stem cell source [combined use of bone marrow and PBSC in studies using the Beijing protocol (12, 14, 103), use of either PBSC or bone marrow in studies using PTCY (3) or *ex vivo* TCD (35)], some trends are becoming evident. An explanation for the consistently higher rates of high-grade aGvHD and extensive cGvHD observed with the Beijing protocol might be the average higher T-cell content in the graft (median 1.5 × 10^8^ T cells/kg bodyweight) (103) associated with the combined use of PBSC and bone marrow as the stem cell source.

The number of T cells in the graft is crucial in the hHSCT setting, and target cell doses have been modified continuously over recent years to optimise the balance between desired graft-vs.-leukaemia effect and unwanted GvHD. Maximum cell doses considered to be safe in terms of avoidance of severe GvHD range between 2.5–5 × 10^4^/kg recipient bodyweight, in association with double pharmacological immunosuppressive therapy (mainly tacrolimus/cyclosporine A combined with mycophenolate mofetil) (104).

The graft source has a significant impact on the occurrence of GvHD: with PTCy both bone marrow and PBSCs are used as the stem cell source. Bone marrow grafts have been shown to bear a lower risk of GvHD but a higher risk of graft failure vs. PBSC grafts (105, 106). Use of PTCy seems to have a satisfactory effect on the prevention of aGvHD but is less effective in preventing chronic forms of GvHD (79). The protective effect of *ex vivo* TCD when compared with PTCy is most evident regarding cGvHD rates (Table 2). Whilst cGvHD in adult transplantation is more often tolerated, in children higher grade cGvHD rates are hardly acceptable, considering the significant impact on quality of life over the lifetime of long-term survivors. A small residual quantity of TCRαβ cells in a T-cell-depleted graft may be the cause of mild aGvHD but a high number of γδ T cells in association with this assures near absence of severe acute or cGvHD (107).

Thus, currently the *ex vivo* TCD platform remains the favourable hHSCT platform with regard to GvHD rates. Particularly in children, cGvHD rates should be close to 0% to best guarantee satisfactory long-term quality of life.

## Post-Transplant Infections

In the hHSCT setting using PTCy (113, 61, 75–78, 81, 99, 104, 106, 107, 113), *ex vivo* α/β TCD (11, 36, 42, 74, 102, 103), and Beijing protocol (7, 16, 63, 66), no definite conclusions regarding the burden of infectious complications can be concluded using currently published data since not all authors report bacterial, viral and fungal infection prevalence, costs, or prolonged length of stay or mortality due to infectious complications in their cohorts. This may be due to the retrospective nature of most studies.

Nevertheless, viral complications are the most frequently reported infectious complications following HSCT but due to different monitoring and pre-emptive treatment strategies among groups (e.g., use of antigenaemia vs. polymerase chain reaction for CMV monitoring) the data between studies are not comparable. This is well-exemplified by the striking differences in the prevalence of CMV viraemia—generally the most common infectious complication reported—between cohorts, with some cohorts having zero cases (76), and some having high prevalence (76.5%) (101). There is an urgent need for more data and standardisation on reporting of infectious complications following HSCT.

## Cost-Effectiveness

At first sight, the PTCy platform seems to be by far the more economic choice of platform for hHSCT than *ex vivo* TCD, with costs of $100 for cyclophosphamide vs. ~$13,000 for graft processing in TCD (45). However, upon closer inspection those differences might be resized. The higher incidence of complications such as veno-occlusive disease, GvHD and haemorrhagic cystitis observed with PTCy need to be considered. Treatment of veno-occlusive disease requires obligatorily hospitalisation for several weeks with substantial costs for pharmacological treatment. Acute and chronic GvHD both lead to prolonged immune suppression and often the need for extracorporeal photopheresis or other interventions with further expenses. In the *ex vivo* TCD setting, potential viral infections with further hospitalisation might further increase total costs. Therefore, besides initial HSCT costs, the long-term management of patients and subsequent expenses need to be considered when aiming for a realistic calculation. However, no formal cost-effectiveness analysis has been performed so far. Economic considerations in the literature, where available, are mainly of approximative character.

Apart from financial aspects, it is evident that the *ex vivo* TCD approach requires sophisticated laboratory facilities and highly specialised knowledge.

A prospective study evaluating the optimal hHSCT strategy in children with acute leukaemia should integrate a cost analysis in order to answer the question of cost-effectiveness. This is essential for the establishment of a standard-of-care approach in a time when increasing restrictions are being placed on healthcare systems worldwide.

## Conclusions, Future Directions, and Open Questions

While hHSCT was previously considered as an alternative option only for those patients without suitably matched donors, it is now being increasingly recognised as an equally feasible option in certain scenarios. In comparative trials using hHSCT in conjunction with the Beijing protocol, hHSCT yielded comparable results in terms of GvHD rate and leukaemia-free survival compared to MUD transplants (66). Another study even suggested better leukaemia control after hHSCT compared to MSD transplantations (67, 70). Bertaina et al. showed in a large retrospective analysis the non-inferiority of TCRαβ/CD19 depleted hHSCT compared to MUD transplants in terms of GvHD- and leukaemia-free survival (35). Thus, substitution of MUD donors by haploidentical family donors is the subject of ongoing scientific research (35, 108). As methods of hHSCT continue to be further optimised, it is conceivable that in the coming years hHSCT will become the method of choice for transplantation of children with acute lymphoblastic leukaemia. However, these data have to be verified in a prospective trial, in which long-term quality of life should be a secondary endpoint. The very low rates of chronic GvHD after TCD hHSCT will certainly impact on this outcome parameter. Furthermore, as was highlightened in the recent COVID-19 pandemic, the advantages of an immediately available donor who can be prepared in the same centre as the patient with no challenging and potentially limiting transfer logistics, add to the attractivity of this approach.

Few studies are available so far comparing the different platforms currently applied for hHSCT in children with ALL. When attempting to perform comparative analyses of different outcome parameters such as OS, relapse, infections or GvHD, one major obstacle is that most cohorts have heterogenous patient populations, include broader populations of malignant disease than just ALL, and sometimes include non-malignant transplant indications as well. Conditioning regimens used in these trials were highly heterogenous, rendering it difficult to perform an adequate analysis focussed on ALL.

Outcome reports do not unequivocally show the superiority of PTCy platforms vs. *ex vivo* TCD or vice versa. 2- and 5-year OS of 65 and 68%, respectively, were observed in paediatric patients transplanted with PTCy and *ex vivo* TCD (35, 60). The relatively new Beijing protocol—which is commonly used in Asia—is associated with higher rates of OS and LFS than *ex vivo* TCD or PTCy with rates of up to 80% for both parameters (15); however, the price is significantly higher incidences of aGvHD and cGvHD. Particularly in children, the avoidance of cGvHD should be a major concern when choosing a transplant regimen, especially when performed in CR1, so that a satisfactory quality of life can be achieved lifelong.

Historical concerns of uncontrollable infections with the *ex vivo* TCD platform are no longer reflected in “real-world” clinical practise due to improvements in methodology. Rapidly evolving and increasingly sophisticated graft engineering with adoptive immunotherapy is an excellent tool to induce efficient virus-specific responses, thus facilitating the good control of infectious complications. Moreover, the GvHD profile of *ex vivo* TCD is significantly advantageous when compared to PTCy; the advantage is even more accentuated when compared with the Beijing protocol.

A common “pro” argument for the PTCy platform is the apparently much lower economic burden, with >100 times lower immediate HSCT-related expenses. However, potential (and frequent) costs during follow-up for hospitalisation and pharmacological treatment due to veno-occlusive disease, GvHD, prolonged immunosuppression and other complications need to be taken into consideration and might, in the end, level the differences.

Restricted access to the laboratory facilities which are indispensable for the TCD approach are still a limiting factor for more widespread use of this technique.

A factor which is not yet reflected in any of the available studies is the impact of the immediate availability of a haploidentical donor for a high-risk malignancy. For realisation of a MUD transplantation several weeks of preparation are required for donor search, confirmatory typing, apheresis scheduling and shipment. In contrast, preparation of a haploidentical donor can be performed almost immediately with little delay. This gain of several weeks might be decisive for a high-risk leukaemia patient, reduce the necessity for bridging therapies and improve transplant outcome.

In conclusion, a prospective clinical trial comparing hHSCT (applied with the three described methodologies) with conventional MUD HSCT is indispensable for answering the open question whether one these donor sources gives superior results in children with ALL and how the different hHSCT techniques compare to each other. Secondary endpoints of such a trial should include cost effectiveness and long-term quality of life. Since no transplant centre will have sufficient experience with all three hHSCT techniques it will be pivotal to conduct this trial internationally with stringent inclusion criteria. International ALL study groups will have to cooperate in order to harmonise the trial design, recruit enough transplant centres with dedicated expertise and allow recruitment of a sufficiently large patient population. Results of such a trial would certainly change the landscape of HSCT in children with ALL.

## Author Contributions

All authors made substantial contributions to the conception or design of the work, drafted the work or revised it critically for important intellectual content, approved the version to be published, and agreed to be accountable for all aspects of the work in ensuring that questions related to the accuracy or integrity of any part of the work are appropriately investigated and resolved.

## Conflict of Interest

The authors declare that the research was conducted in the absence of any commercial or financial relationships that could be construed as a potential conflict of interest. The handling editor declared a shared consortium with the authors at time of review.

## Publisher's Note

All claims expressed in this article are solely those of the authors and do not necessarily represent those of their affiliated organizations, or those of the publisher, the editors and the reviewers. Any product that may be evaluated in this article, or claim that may be made by its manufacturer, is not guaranteed or endorsed by the publisher.
